# ELNdataBridge: facilitating data exchange and collaboration by linking Electronic Lab Notebooks via API

**DOI:** 10.1186/s13321-025-01024-1

**Published:** 2025-05-26

**Authors:** Martin Starman, Fabian Kirchner, Martin Held, Catriona Eschke, Sayed-Ahmad Sahim, Regine Willumeit-Römer, Nicole Jung, Stefan Bräse

**Affiliations:** 1 Institute of Biological and Chemical Systems, Functional Molecular Systems (IBCS-FMS), Karlsruhe Institute of Technology, Kaiserstraße 12, 76131 Karlsruhe, Germany; 2 Karlsruhe Nano Micro Facility (KNMFi), Kaiserstraße 12, 76131 Karlsruhe, Germany; 3 Institute of Metallic Biomaterials, Helmholtz-Zentrum Hereon, Max-Planck-Straße 1, 21502 Geesthacht, Germany; 4 Institute of Membrane Research, Helmholtz-Zentrum Hereon, Max-Planck-Straße 1, 21502 Geesthacht, Germany; 5 Institute of Organic Chemistry, Karlsruhe Institute of Technology, Kaiserstraße 12, 76131 Karlsruhe, Germany

**Keywords:** Electronic Lab Notebook, FAIR data, Interoperability, Chemistry, Materials sciences

## Abstract

Electronic Lab Notebooks (ELNs) have become indispensable tools for modern research laboratories, facilitating data management, collaboration, and documentation of scientific experiments. However, the proliferation of diverse ELN platforms poses challenges for researchers who need to seamlessly exchange data between different systems. In this paper, we present ELNdataBridge, a novel server-based solution designed to address this challenge by providing a flexible adapter for interfacing and synchronising data between disparate ELN platforms. ELNdataBridge leverages Python APIs to interact with the underlying data structures of various ELN systems, enabling smooth transfer of information between them. The system offers a user-friendly interface that allows researchers to map and configure the transfer of single values and entry types between different ELNs, thereby facilitating interoperability and data exchange. The suitability and efficiency of the developed software was shown by a first demonstrator, enabling the exchange of data from Chemotion ELN and Herbie, and therewith the connection of information with a focus on chemistry and materials sciences.

**Scientific contribution:** To the best of our knowledge, a method enabling the interoperable exchange of information between different ELNs, as described here, has not yet been reported. Given the increasing number of scientists using ELNs and their reliance on discipline-specific platforms, this work proposes a solution to overcome the current limitations related to ELN interoperability.

**Supplementary Information:**

The online version contains supplementary material available at 10.1186/s13321-025-01024-1.

## Introduction

As essential platforms for the digitalization of modern research laboratories, Electronic Lab Notebooks (ELNs) assist scientists in data management, collaboration, and documentation of scientific experiments, enhancing the reproducibility, sustainability, transparency, and traceability of experiments. Depending on the researchers' needs, ELNs must support various processes and incorporate tools for working in different disciplines. Due to the complexity of requirements as well as the diverse handling and structuring of data, there are different ELNs utilised by researchers in their respective fields [1]. Generic or multidisciplinary ELNs typically provide researchers with significant freedom in data storage methods and process documentation, but often fall short in ensuring the availability of well-annotated and standardized data.

There are also ELNs with specialised functionality and a data structure well adapted to each respective discipline. The standards and processes prescribed by these discipline-specific ELNs can effectively accelerate scientific work, increasing the acceptance of user scientists. They can also ensure FAIR [2] (Findable, Accessible, Interoperable, Reusable) data storage to ensure the direct and long-term reusability of these data and comparability with other sources. Selecting the appropriate ELN software is not trivial, in particular as ELNs are often established by the respective research institutions that have to address the needs of significantly different disciplines and interdisciplinary work [3]. Providing added value to such interdisciplinary work and the support of different disciplines can only be achieved through a combination of ELNs [4] which then raises the question of interoperability between ELNs immediately. Obviously, interoperability software solutions need to ensure data transfer from one ELN environment to another with minimal loss.

Unfortunately, there are currently no standardised protocols for exchanging data between ELNs, creating a large hurdle for data transfer or migration from one system to another [5]. There are various approaches to this issue, assuming that the data structure, contents, and functions of the ELNs cannot be harmonised. Possible paths toward using data in different ELNs could include:

1. Generating data exchange formats that can be interpreted by all ELNs equally.
2. Providing bilateral interfaces between each pair of ELNs that can communicate with each other.
3. Developing converters to convert data from one ELN format to another.
4. Developing external software to multilaterally moderate the interfaces.

In this paper, we focus on the latter option and present a novel server-based solution designed to address the interoperability challenge by providing a flexible adapter for interfacing and synchronising data between disparate ELN platforms.

## Related work

There are already approaches to solve the ELN interoperability problem being advanced by individual providers or consortia, aiming at long-term harmonisation of research data and metadata. The group “The ELN consortium”, involving developers of ELNs from academic institutions, is working towards generating a unified format (*.eln) [6] that can be used by all participating ELNs based on the well-established RO-Crate specification [7, 8]. This approach has a high potential for being successful in offering data exchange solutions in the long run. Also, the ELN consortium improves the FAIRness of data due to continuous extensions of the metadata descriptions in the *.eln file, e.g. by adding further semantic annotations using JSON-LD. However, a file exchange requires high implementation effort for the ELN developers, as the transfer of specific data requires adaptation of the file format for each pair of ELNs, also keeping the format updated to newer ELN versions. Moreover, moving files induces much manual work for the users.

Beyond exchange file formats, there are initiatives that work on the specification of scientific data. SciMesh [9] defines the representation of scientific results in the form of a knowledge graph including physical specimens to facilitate the exchange of information on particular samples from one ELN to another one. Also Bioschemas [10, 11] offer standardised descriptions that can be used to map the content of ELNs to common schemas and ontologies. Being developed for improving the findability of life science records in the web, the types and properties defined by BioSchemas can serve as reference to describe ELN content in a suitable way.

At first sight, employing established standards, i.e. schemas or file formats, appears to be particularly sustainable to achieve ELN interoperability, regardless of the interfaces of the respective software. While this approach for ELN software with less structured content offers satisfactory solutions in the medium term, applying it to very discipline-specific ELNs with a high degree of structure becomes a complex undertaking that cannot currently be completed to satisfaction. The main reason for this is the inability of a schema to cover all specifications for the diverse contents of discipline-specific documentation, which consequently cannot be transferred clearly and in detail from one ELN to another by a data exchange format. In order to develop satisfactory solutions for those discipline-specific ELNs, for example, an Application-Programming-Interface-based (API-based) exchange of information should be considered.

A direct API exchange, with defined communication protocol and standard for objects, would bear the same disadvantage as a file exchange format: every new ELN needs to implement an API with all existing ELNs. Hence, an advantageous solution employs a central server as an exchange hub. To maintain data security, no central database of ELN entries is implemented, but merely moderation of the communication.

## Results and discussion

### Exemplary ELNs for interdisciplinary data exchange

As an exemplary use case, the data exchange between the two ELNs Herbie and Chemotion was explored.

Chemotion [12] is an open-source ELN developed by the Karlsruhe Institute of Technology (KIT) in Germany in collaboration with other partners of the National Research Data Infrastructure for chemistry (NFDI4Chem) [13, 14]. Chemotion is tailored to the needs of researchers in the field of chemistry, providing features to facilitate data management, experiment documentation, the analysis of measurements and analytical data [15], and collaboration. Chemotion aims to streamline the research process in chemistry laboratories by offering tools for recording experimental data, for drawing and processing chemical structures [16, 17], the transformation of proprietary files into open file formats, the management of a chemical inventory, and the organisation of research workflows. As an open-source project, Chemotion allows users to customise and extend its functionality according to their specific requirements. Overall, Chemotion is designed to enhance efficiency, reproducibility, and collaboration in chemistry research through its features tailored to the needs of chemists with an experimental focus. It can be used to seamlessly transfer data into the research data repository Chemotion [18] to publish research data.

Herbie is developed at the Helmholtz-Zentrum Hereon as an open-source and modular ELN to interlink heterogeneous process chains in research [19]. It is ideally suited for labs with fabrication and characterization processes that are standardised or performed repetitively, e.g. for materials sciences. To achieve comparability, quality assurance, and reusability, users enter data in well-structured, pre-defined webforms for each process or entity type. These webforms are automatically generated from ontologies, defined in OWL (Web Ontology Language) and SHACL shapes (Shapes Constraint Language) [20]. Through the integration of ontologies, all inserted data is semantically annotated and the webforms are thoroughly interconnected, reflecting the complete lifecycle of a sample. Thus, all entered information is easily accessible in various contexts, such as in a chronological journal, filtered for the linking entities like projects or equipment, or in a tabular database. Users can create, view, update or delete entries via the graphical web interface or its REST API. File export, file import and the API embed Herbie conveniently into linked-data software ecosystems.

Chemotion and Herbie entail very complementary features, both from a frontend and backend perspective, and thus jointly offer to seamlessly document the entire process chain from the molecular design to the engineering of components and plants. Therefore, a data exchange mechanism between these two ELNs would enable synthetic chemists, materials scientists, and engineers to create an interoperable and rich database. By providing an API, both ELNs fulfil the basic requirements for this endeavour.

### Conceptual mapping of ELN features

The first step for linking ELNs comprises the conceptual mapping of their corresponding content. It is up to the scientists and ELN operators to synchronize only those input entities that are of relevance and to verify the coherence of their meaning. An experienced user of both ELNs is required for this mapping exercise, which could be facilitated by a schema of input variables by the ELN providers. Such schemas for Herbie and Chemotion are provided with the ELNdataBridge code [21].

Mapping the content of one ELN to another—even when covering only parts of interest in a certain data exchange scenario—can be a complex task, as ELNs may have different data structures that may prevent options for a one-to-one mapping of content. Also, the granularity of descriptions referring to certain ELN entities may be quite different, depending on the focus of the ELN’s application and use-cases. As a result, one ELN may have a certain set of information stored in well-defined database tables with values therein most likely correlated to standardised units, whereas the same information is given in textual form in the other ELN. In the worst case, one ELN stores information that is neglected in the other and vice versa.

These schematic conflicts were solved in work described therein for the ELNs Chemotion and Herbie for the use case “Polymer Membrane Post-Modification”. For the given use-case, we considered the relevant content of the selected ELNs, which was covered in Chemotion ELN with the ELN models (input masks) for “reaction” and “sample”, and in Herbie with the ELN models for “post-modification” and “product”. The content of the required ELN models was mapped from one ELN to the other (Figures S1, S2), and conflicts were resolved by different means, e.g. the creation of new input fields (if a lack of information was detected and the need for an adaptation obvious) or converting date-time formats (more details in the SI).

### Architecture of an API-based adapter server—ELNdataBridge

**Fig. 1:**
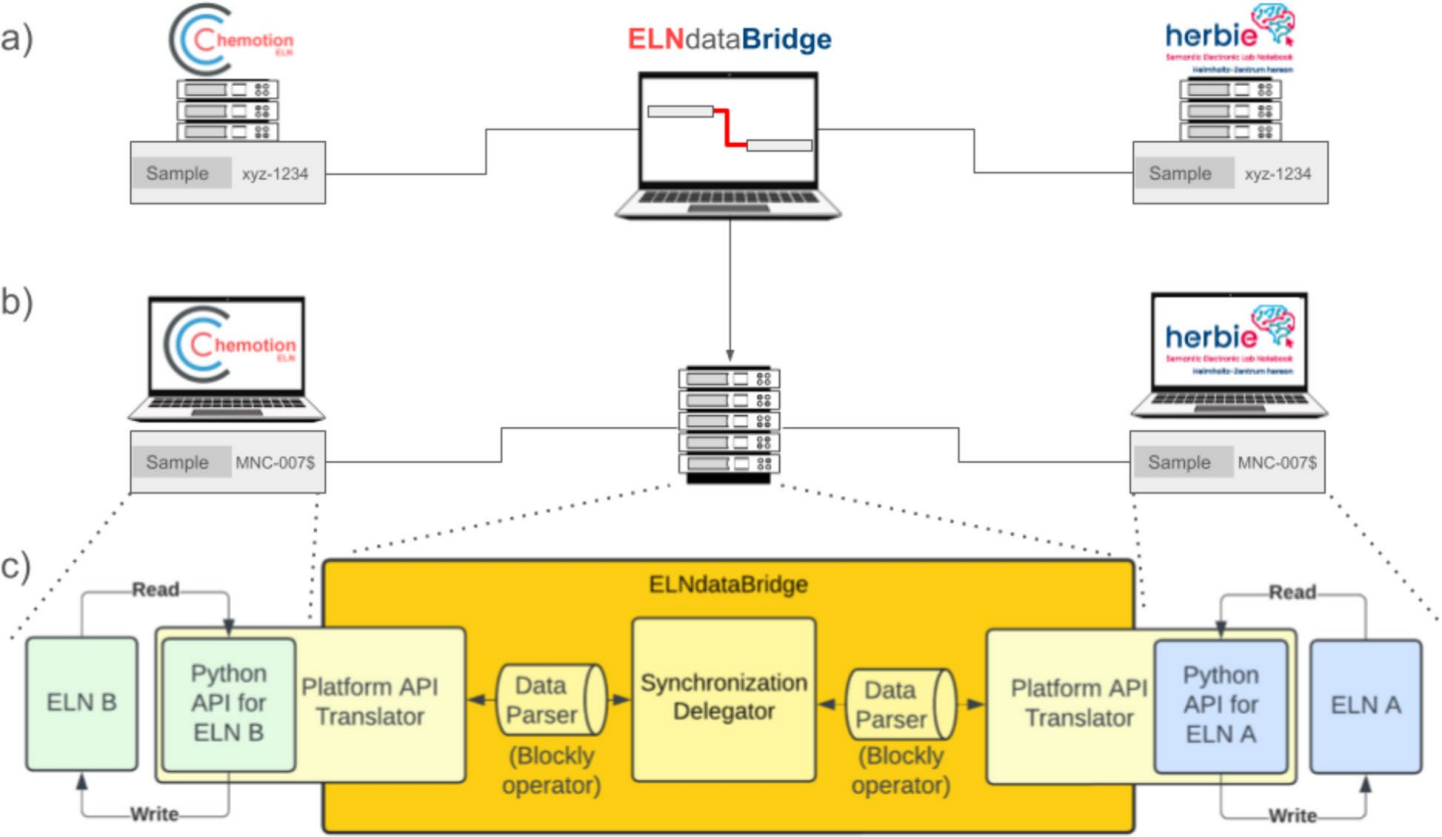
Schematic description of the main components and actions of ELNdataBridge: **a** set up of the data transfer with the user interface; **b** operation and application; **c** schematic representation of the backend architecture

The main component to enable the generic connection of two (or more) ELNs is constituted by an API-based adapter server (ELNdataBridge). ELNdataBridge leverages Python APIs to interact with the underlying data structures of various ELN systems, enabling the mapping of information and thereafter the seamless transfer of information between them. The ELNdataBridge covers therefore two main workflows:

1. Creation of the sync model: The identification of available content (source field) in a selected ELN, the search for corresponding content in the desired partner ELN (target field) and the mapping of both via the client of ELNdataBridge (Fig. 1a), and vice versa.
2. Execution of the sync model: The synchronisation of the available content in both ELNs for the mapped information according to predefined rules. This process includes the monitoring of changes in both ELNs and the continuous synchronisation of the content of both (Fig. 1b).

To enable the two workflows, the ELNdataBridge consists of a frontend (= User Interface) and a backend (= business logic for the synchronisation) component:

The *User Interface (UI)* is a common server-client application that can be used to configure the ELN-to-ELN data exchange. This includes the registration process of ELNs to ELNdataBridge and the user-friendly mapping of the content of two ELNs, driven by a data parser in the backend, without expecting coding skills from the users (Fig. 1a). It is based on Django, a robust Python framework. Due to the specifications of SimpleDomControl, a Python/Javascript framework and Django extension, the architecture of the system was developed strictly according to a Model-View-Controller pattern. As a data storage component, a SQL database is available. Django enables a database-independent design, capable of interfacing with various SQL databases. However, by default, it is configured to use a PostgreSQL database.

The *business logic of the synchronisation* is provided for each ELN via their respective platform API and enables seamless communication between the system and the ELNs, based on a delegation pattern. The so-called synchronisation delegator is provided as a moderator of communication between the platforms (Fig. 1c). The delegator performs the following tasks in each process step: it finds all entries from both platforms that are mapped for synchronisation in the UI. From the entries found, the delegator identifies the matching pairs of entries between the two platforms involved. Then, it determines whether values have changed since the last synchronisation process and therefore need to be transferred to the respective complement. All communication to the actual ELN APIs is delegated by the delegator to a delegate. A delegate must be provided for each participating platform. These delegates must implement a so-called platform API translator. To fulfil the requirements of the interface, the delegate must implement a set of Python functions that handle reading and writing individual entries, requesting all entries of a certain type, as well as listing all types that are available.

### Establishing ELN-to-ELN data mapping

**Fig. 2:**
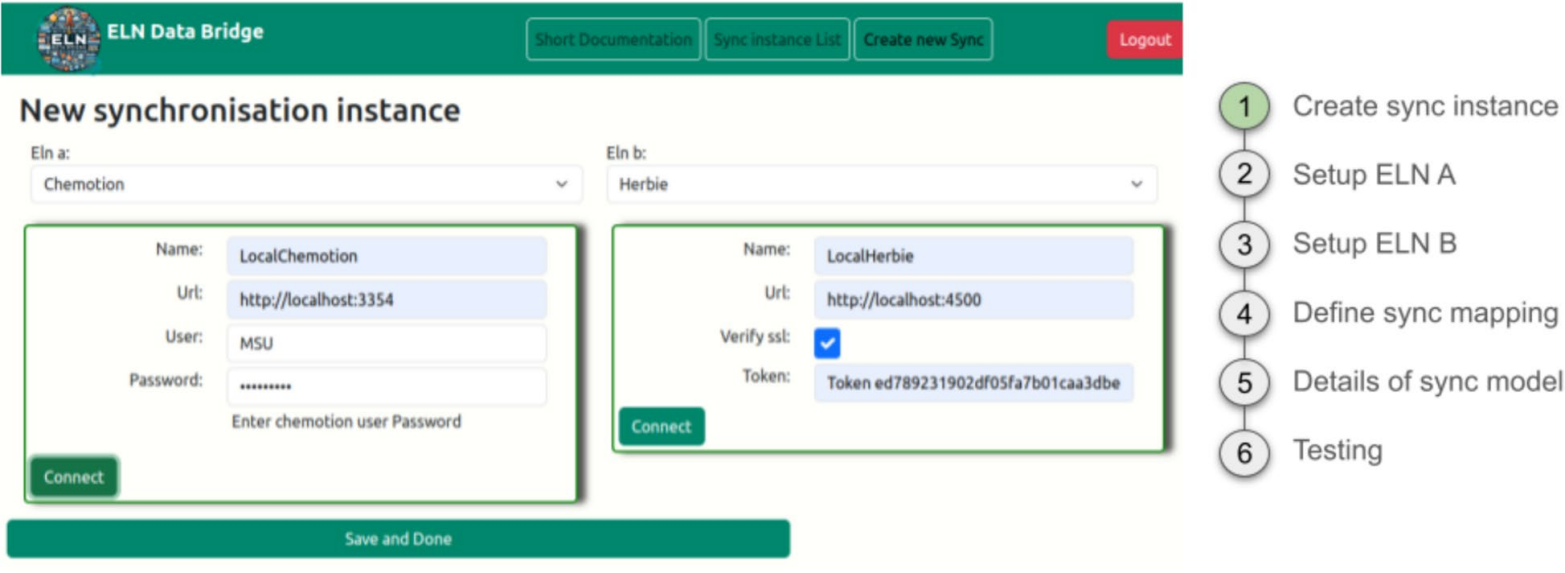
Configuration of a synchronisation interface between local ELN instances of Chemotion and Herbie. Screenshot taken from ELNdataBridge; reworked/cut to enhance clarity (full screenshot see SI Figure S4)

The establishment of the ELN-to-ELN mapping process in ELNdataBridge needs the customization of the planned data exchange using the mapping of the ELNs’ content as described before. The UI allows researchers to configure the required aspects of the ELN-to-ELN data exchange and to map and configure the transfer of single values and entry types between different ELNs. In a first step, a connection between the ELNs is established by the generation of a new synchronisation instance. This requires the entering of necessary information such as the name, URL, and token or password assigned per ELN instance and user of the instance (Fig. 2).

**Fig. 3:**
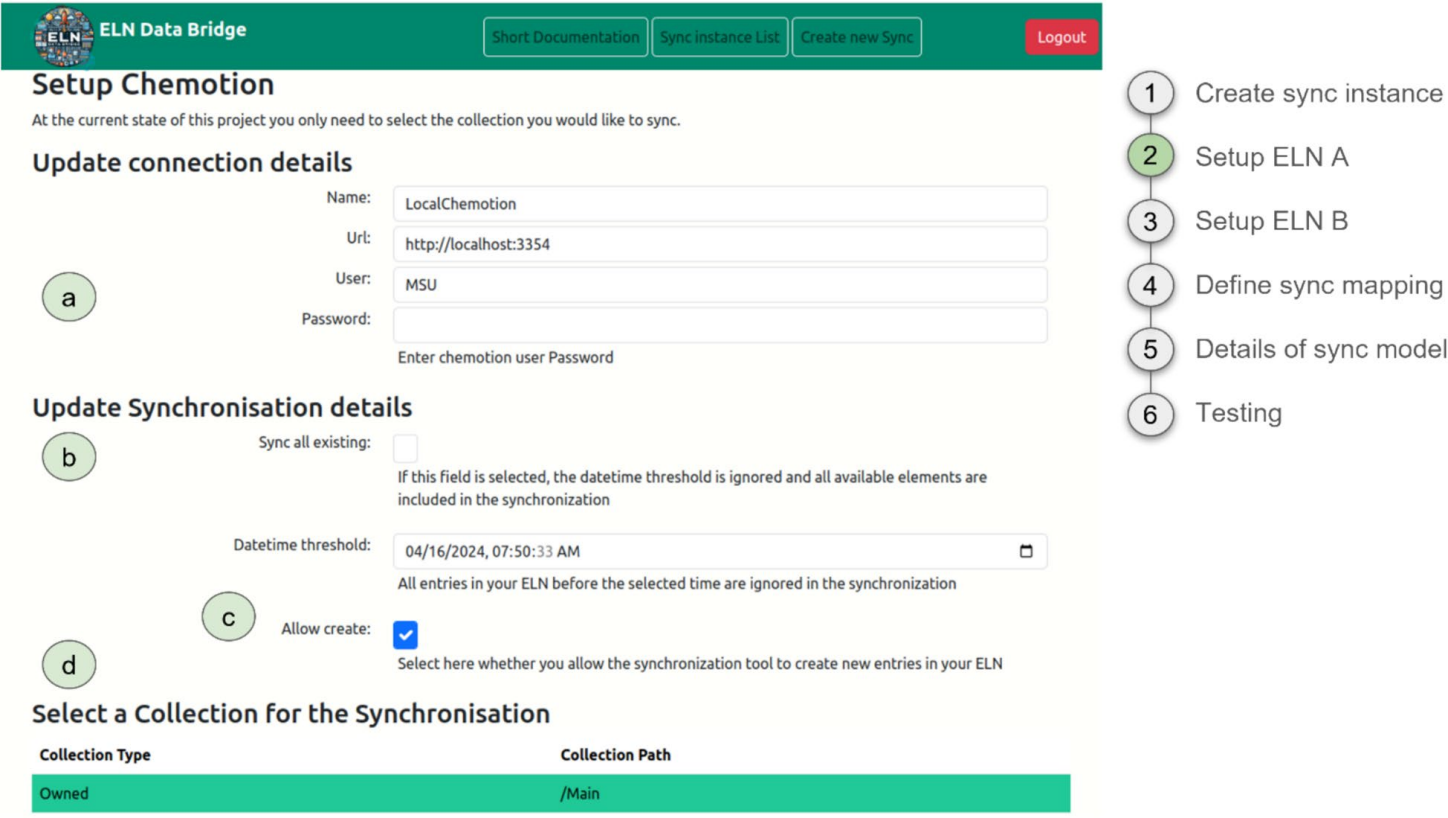
Settings of the synchronisation parameters for Chemotion. **a** Settings of the connection. **b** Settings of how existing entries should be handled. **c** Decision about the creation of new entries. **d** Selection of the collection as a structural element in Chemotion for thematically related datasets (for Herbie see SI Figure S5)

In the next steps 2 and 3, the setups of ELN A, e.g. Chemotion, and ELN B, e.g. Herbie, have to be configured (Fig. 3, exemplarily for ELN A). The updating of connection details and the configuration of essential properties are critical steps to ensure a smooth synchronisation process in ELNs. The choice of properties encompasses decisions such as whether to include all existing entries in the synchronisation or only those last updated after a specific timeframe. Additionally, consideration must be given to whether the automatic generation of new entries should be permitted within the ELN environment. Given that projects and activities in Chemotion are structured within collections, synchronisation hinges on choosing one within your user account to establish the initial link between ELNs. In contrast, Herbie lacks a comparable feature; hence, no additional outline element necessitates selection.

**Fig. 4:**
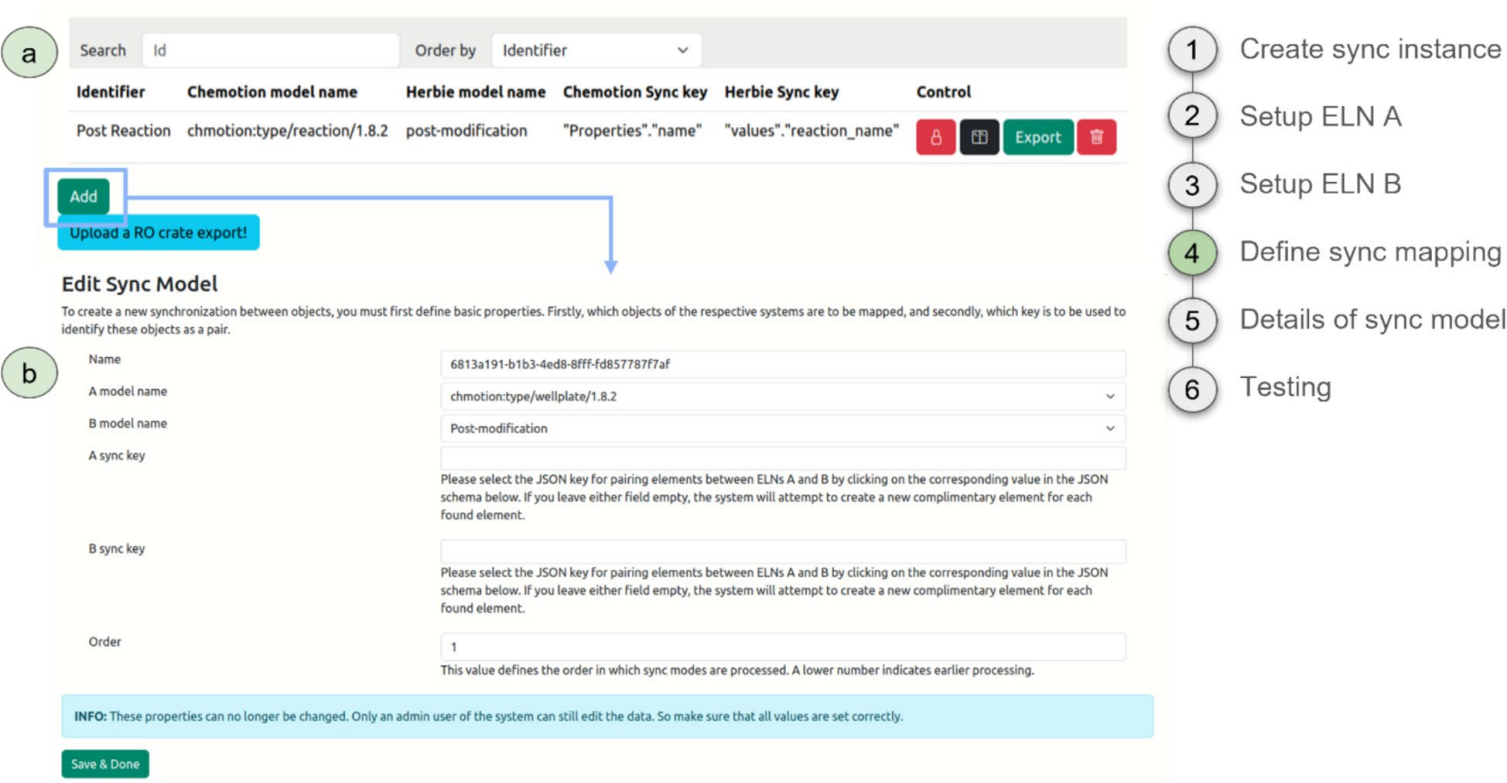
Defining the main properties and pairing of the sync model. **a** Exemplary list of sync models with two entries. The sync model on top synchronises Chemotion’s *reaction* input masks with Herbie’s *post-modification* input masks. The sync keys used for the pairing before synchronisation are *properties.name* for Chemotion and *values.reaction_name* for Herbie. **b** This definition and pairing definition and pairing can be edited

After setting up the general mapping framework with steps 1–3, the Sync Model Manager (Figure S3) of ELNdataBridge allows defining the main properties and pairing settings (Fig. 4) by either the creation of new synchronisation models or the management of existing ones. Each sync model requires a unique identifier, which is a human-readable name, and the names of the two ELN models (input masks) that need to be synchronised (Fig. 4b). It is imperative that the desired model is selected with the correct version to be synchronised in order to avoid synchronisation errors after an update of the system (see also SI chapter 4 for version dependencies related to schema changes). Additionally, it’s crucial to set the keys for both ELN models. The system uses these keys to identify and match pairs to ensure the accuracy of the synchronisation process.

A detailed configuration of the sync model for the ELN mapping can be set in forms that guide the user through the required information, supporting a customizable synchronisation process. Most of the fields in the user-friendly form rely on a click-and-select mechanism by dropdowns or a selection from the JSON representation of the objects (input fields) displayed below the form.

**Fig. 5:**
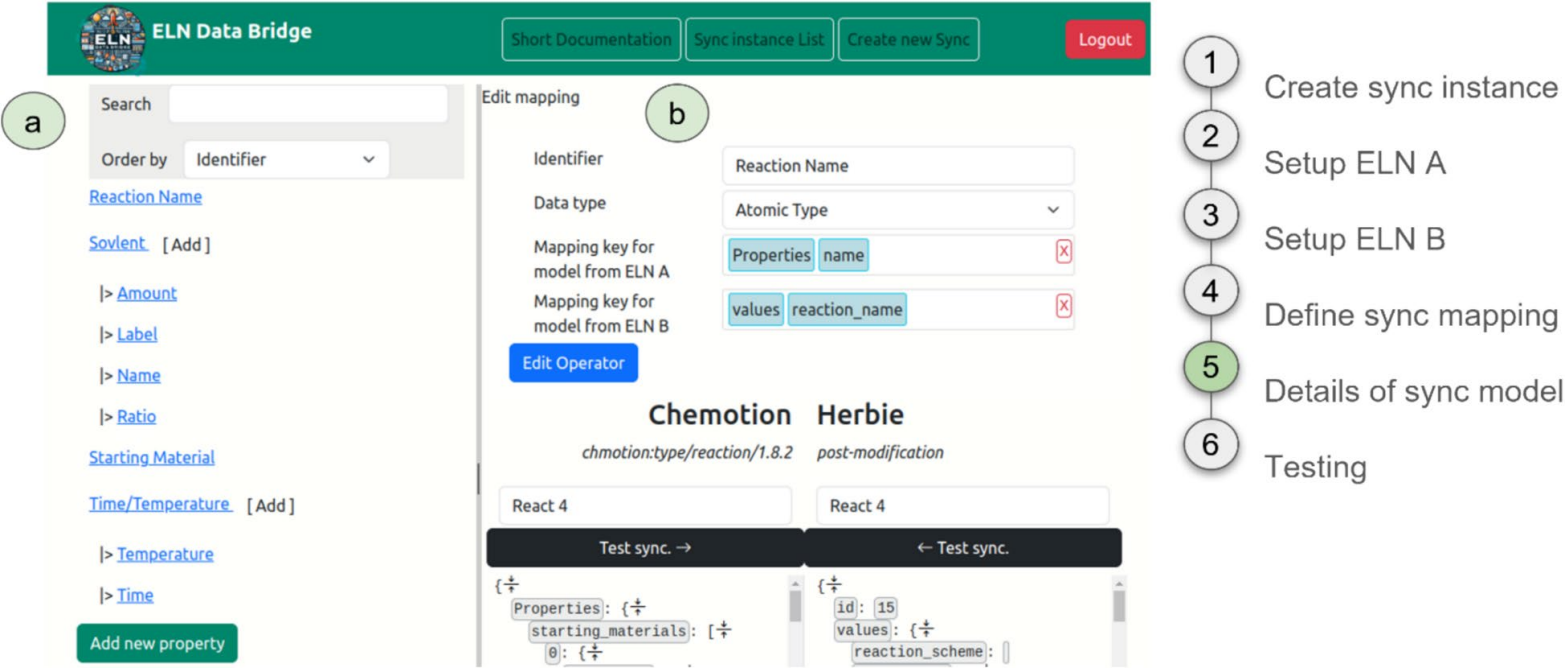
Setting the mapped objects (input fields) of the sync model. **a** This sync model contains ten mappings from *Reaction Name* to *Time*. **b** Each mapping can be edited: The *Reaction Name* mapping uses the *Atomic* data type and synchronises the objects *properties.name* from Chemotion and *values.reaction_name* from Herbie, as set by the mapping keys

The list of mapped objects within a sync model acts as the focal point for configuring mappings between ELN systems (Fig. 5). From this section, users can access the central mapping settings, each comprising the following components:

1. Identifier: A unique identifier assigned to each mapping of objects.
2. Data type: A descriptive explanation accompanying each mapping of objects, outlining the functionality of the transfer operator.
3. Key: A navigational path enabling access to the corresponding key within the JSON representations of both ELNs.

When setting the actual values in the sync model’s mapping form, the only property that requires manual input is the identifier, as in the model pairing form. Also as above, the mapping keys can be set using the JSON representation below the form. Moreover, it is important to set the data type. Users are presented with two data types to choose from: array or Atomic value. Entries of type *Array* do not possess properties or operators; instead, they solely contain submappings which are applied to each entry within the list. The second type *Atomic value* is used to directly synchronise the actual entries of the ELN objects (input fields). By default the mapping by Atomic value simply copies a value from one ELN to the other, leaving the data type unchanged. In instances where there is a necessity to modify the value or data type, manipulation of the transfer operator is possible. This manipulation can be executed using the Blockly web library, developed by Google. Blockly offers a visual programming interface, employing drag-and-drop blocks to illustrate programming concepts like variables, logical expressions, loops, and more. Despite its simplicity, Blockly proves highly effective in implementing data type transformations or even altering actual values in such cases.

Once all configurations have been set, the pairing and mapping can be tested. The test consists of two simulated synchronisation processes which can be triggered independently of each other. One reads information from the JSON representation of the object of ELN A and writes it into the object of ELN B. In the same way, the simulated synchronisation process can be performed the other way around, testing the writing process from objects of ELN B into ELN A. After the execution of the test, all changes (of only one object) based on the current mapping are plotted in a summarising dialog (SI Figure S6).

### The synchronisation process

After successful testing, the synchronisation process should be started manually once in the main menu to be able to monitor the progress of the sync process via process logs. After this, a cronjob (schedules tasks to run them periodically at fixed intervals in order to automate regular, repetitive jobs) triggered routine enables the synchronisation with *Activate Autosync*. Synchronisation progress, i.e. the server activity and the history of all processes can be monitored in real time in a list in the main menu (SI Figure S7). The changes to each individual object can be tracked via a link in the logs (SI Figure S8).

**Fig. 6:**
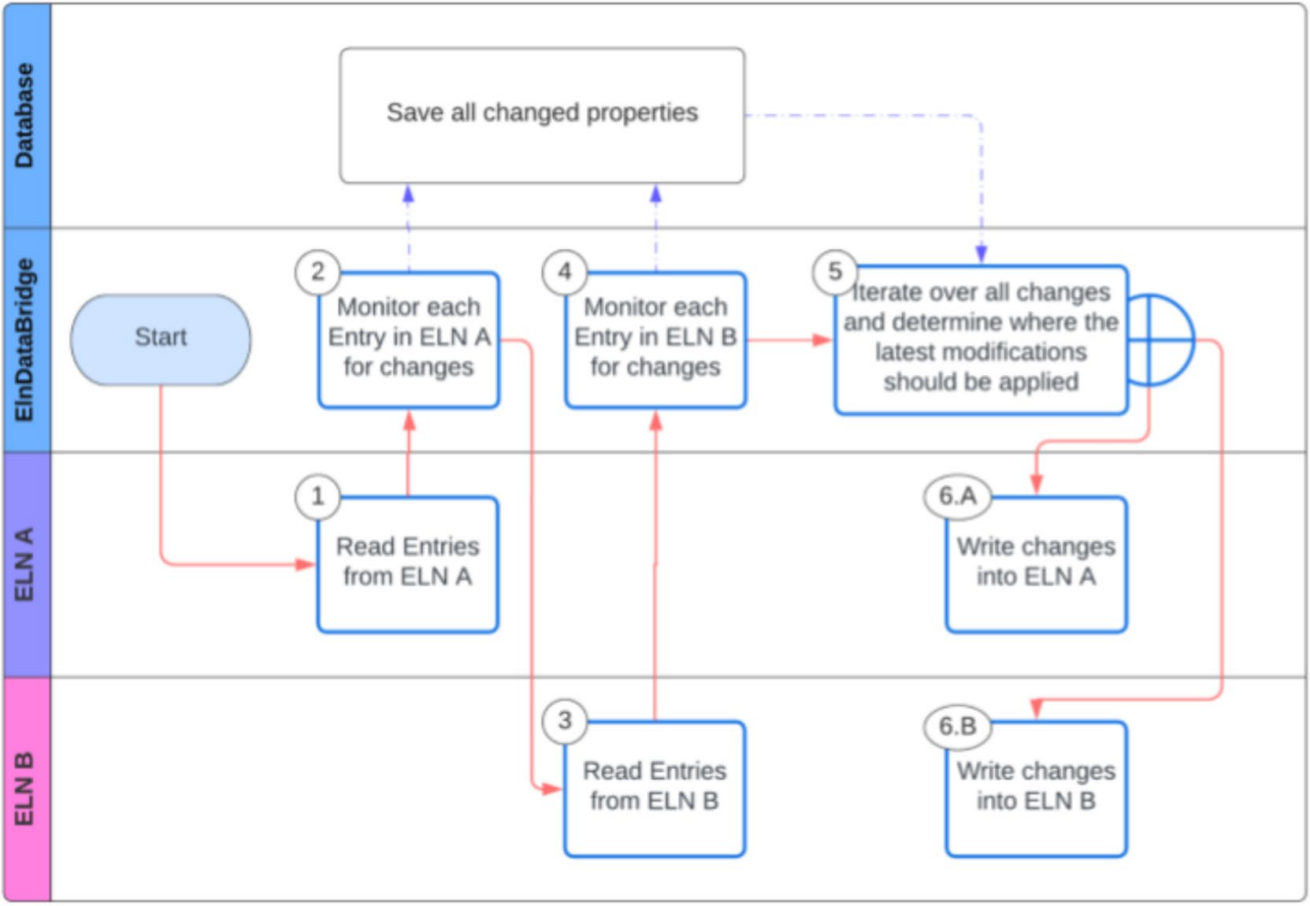
Schema of the workflow that allows the synchronisation of the database content of two ELNs A and B

The actual process of synchronisation is illustrated in a simplified form in Fig. 6. All entries are read one after the other, first from ELN A (Fig. 6, step 1), then from ELN B (Fig. 6, step 3). For each entry, a check is made for changes of the values configured by the mapping specifications (see Fig. 5) since the last synchronisation process (Fig. 6, steps 2 and 4). If so, a corresponding data entry is made. As soon as all entries have been read, the process searches for the availability of complementary entries in the target ELN (Fig. 6, step 5). In case of missing entries, an attempt is made to create a new one if the corresponding settings allow it (Fig. 3c). The new values are now transferred to the entries (Fig. 6, steps 6.A and 6.B) found (or created) according to the mapping specifications (see Fig. 5). In this step, the transfer operator is used to adapt the data to the target system, the latest updated version of two pairing entries will overwrite all existing values in its partner in cases where the settings define that all or parts of the entries are to be synchronised. Complex data and complex data types can be synchronised as well. However, linked entries in the current system can become a challenge as linking existing entries as part of a synchronisation process is not a trivial problem and ELNdataBridge offers only limited options to solve this challenge.

### Use-case “chemical post-modification of membranes”

Exemplarily, we will demonstrate the capability of ELNdataBridge by applying it to metadata of the chemical post-modification of polymer membranes. In this use-case, the membrane generation [22] is described in Herbie and fundamental properties of this membrane must be transferred to Chemotion before information about the chemical treatment can be entered. Subsequently, the chemical reactions of molecular substances with a polyacrylonitrile membrane [23–25] are recorded in Chemotion, while further performance characterization and the assembly of membrane modules will be documented in Herbie. Hence, the metadata of the chemical experiments must be transferred from Chemotion to Herbie for future reference and dataset assembly, creating a full picture of the entire process chain for the researchers.

**Fig. 7:**
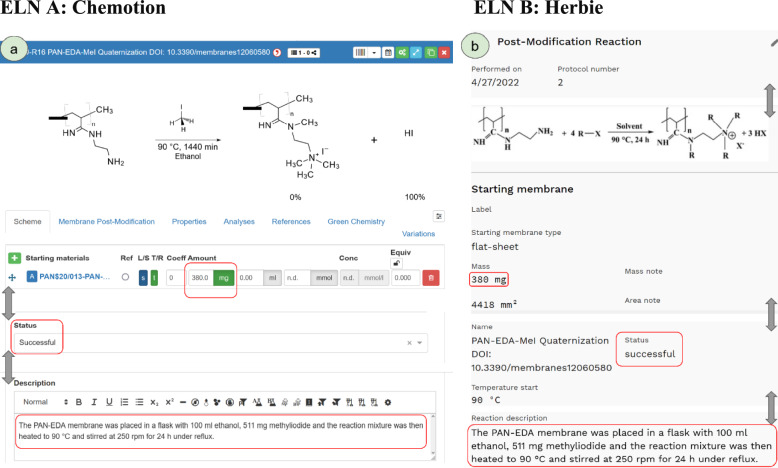
**a** Chemotion reaction template with highlighted changes. **b** Herbie post-modification form after synchronisation with the equivalent changes

Prior to the conducting the chemical experiment, the existing membrane “PAN$20/013-PAN-EDA-01;” is generated as a “product” in Herbie by noting fabrication details, and automatically created as a “sample” in Chemotion via the ELNdataBridge synchronisation. After the post-modification experiment, the filled-in reaction template in Chemotion (Fig. 7a) contains parameters such as employed weight of the membrane (integer), a description of the reaction conditions (free-text) and status of the reaction (selection from list). Selecting the collection and reaction in Chemotion, followed by synchronisation, creates all data in Herbie. Subsequent changes to the three aforementioned parameters are updated in Herbie via ELNdataBridge (Fig. 7b). Thus, ELNdatabridge enables a seamless, quick and low-effort metadata transfer of chemical reactions between Herbie and Chemotion. This provides scientists and ELN users a simple means to transfer data between the ELN platforms.

### Expanding ELNdataBridge to ELNs

To interact with ELNdataBridge, the fundamental requirement for an ELN is the existence of an API. As the architecture of the ELNdataBridge aims to minimise the development effort on the side of the ELNs, their platform API translator (Fig. 1) merely needs to be composed once. Obviously, the effort to synchronise two ELN models (input masks) depends on their extent and number of objects. Table S1 in the SI compiles an overview of the integrability of open-source ELNs into ELNdataBridge.

## Conclusion

In this study, it was shown that interoperability between different Electronic Laboratory Notebooks (ELNs) designed for different scientific disciplines and with a different data structure can be achieved by moderating the communication and synchronising data between the ELNs. The proposed API adapter system coined ELNdataBridge requires ELNs to follow a defined registration principle for translation. Once the required definitions and settings are introduced, the automatic data exchange implies minimal manual effort for users. Key features of the ELNdataBridge development include (1) a high degree of adaptability by utilising Python APIs to interface with a wide range of ELN platforms that support Python integration, (2) a graphical user interface that enables users to intuitively map data objects between different ELN systems, providing fine-grained control over the synchronisation process, (3) well-defined synchronisation processes ensuring that changes made in one ELN are promptly reflected to connected systems, and (4) the option to extend the system continuously to handle large volumes of data efficiently, ensuring scalability to meet the needs of diverse research environments. Through a series of case studies and practical demonstrations, we illustrate the suitability and versatility of ELNdataBridge in facilitating seamless data exchange between popular ELN platforms. By integration of other ELNs that were identified as suitable candidates, the contrived mapping schemes can be used towards the development of multi-discipline standards for the description of frequently used entities, leading to harmonization of descriptions. We believe that ELNdataBridge represents a valuable contribution to the research community, offering a robust solution for addressing the interoperability challenges associated with heterogeneous ELN environments.

## Supplementary Information

Supplementary Material 1.

## Data Availability

Availability of source code and software: The software that was produced in this project (core ELNdataBridge component) is available under open-source licenses on the Helmholtz Cloud (https://codebase.helmholtz.cloud/eln-diy-meta/labdatabridge.git) and Zenodo [26]. The work based on the ELN’s APIs of Chemotion and Herbie is available as an Open Source on codebase.helmholtz.cloud and Zenodo: Chemotion API: https://codebase.helmholtz.cloud/eln-diy-meta/chemotionapi, 10.5281/zenodo.12569190, Herbie API:https://codebase.helmholtz.cloud/eln-diy-meta/herbieapi, 10.5281/zenodo.12569421. Availability of demo video: 10.5281/zenodo.13150464. User guide/documentation: https://elndatabridge.readthedocs.io. Requirements: The requirements to install the Chemotion ELN are described in detail in the documentation of Chemotion ELN (https://www.chemotion.net/docs/eln/install_configure). Likewise, instructions on how to setup Herbie can be found at https://codebase.helmholtz.cloud/hereon-mb/herbie#try-out. The work described here was tested to be compatible with versions of Chemotion ELN v.1.8.2 and v.1.9.2. (10.5281/zenodo.1054134) and Herbie ELN v0.1 (10.5281/zenodo.12205429).

## Abbreviations

API: Application Programming Interface
ELN: Electronic Lab Notebook
FAIR: Findable, Accessible, Interoperable, Reusable
JSON(-LD): JavaScript Object Notation (for Linked Data)
OWL: Web Ontology Language
REST: Representational State Transfer
SHACL: Shapes Constraint Language
SQL: Structured Query Language
UI: User Interface
