# Supplementary material for: ELNdataBridge: facilitating data exchange and collaboration by linking Electronic Lab Notebooks via API
Source: J Cheminform. 2025 May 26;17:86. doi: 10.1186/s13321-025-01024-1 (PMC12107819; doi:10.1186/s13321-025-01024-1)
Supplement: Supplementary file 1 — Supplementary Material 1. [file 13321_2025_1024_MOESM1_ESM.pdf]

Supplemental Information

ELNdataBridge: Facilitating Data Exchange and Collaboration by Linking Electronic Lab Notebooks via API

1. Conceptual Mapping of ELN features ... 2
2. User Interface ... 4
3. Setup details ... 7
   3.1. Details on building the pairing and mapping ... 7
   3.2. Details on testing ... 7
   3.3. Details on synchronisation ... 8
4. Dependencies arising from changes of the schemas ... 10
5. How to include a new ELN to ELNdataBridge ... 11
6. Classification of Open Source ELNs referring to the suitability for ELNdataBridge ... 12

1. Conceptual Mapping of ELN features

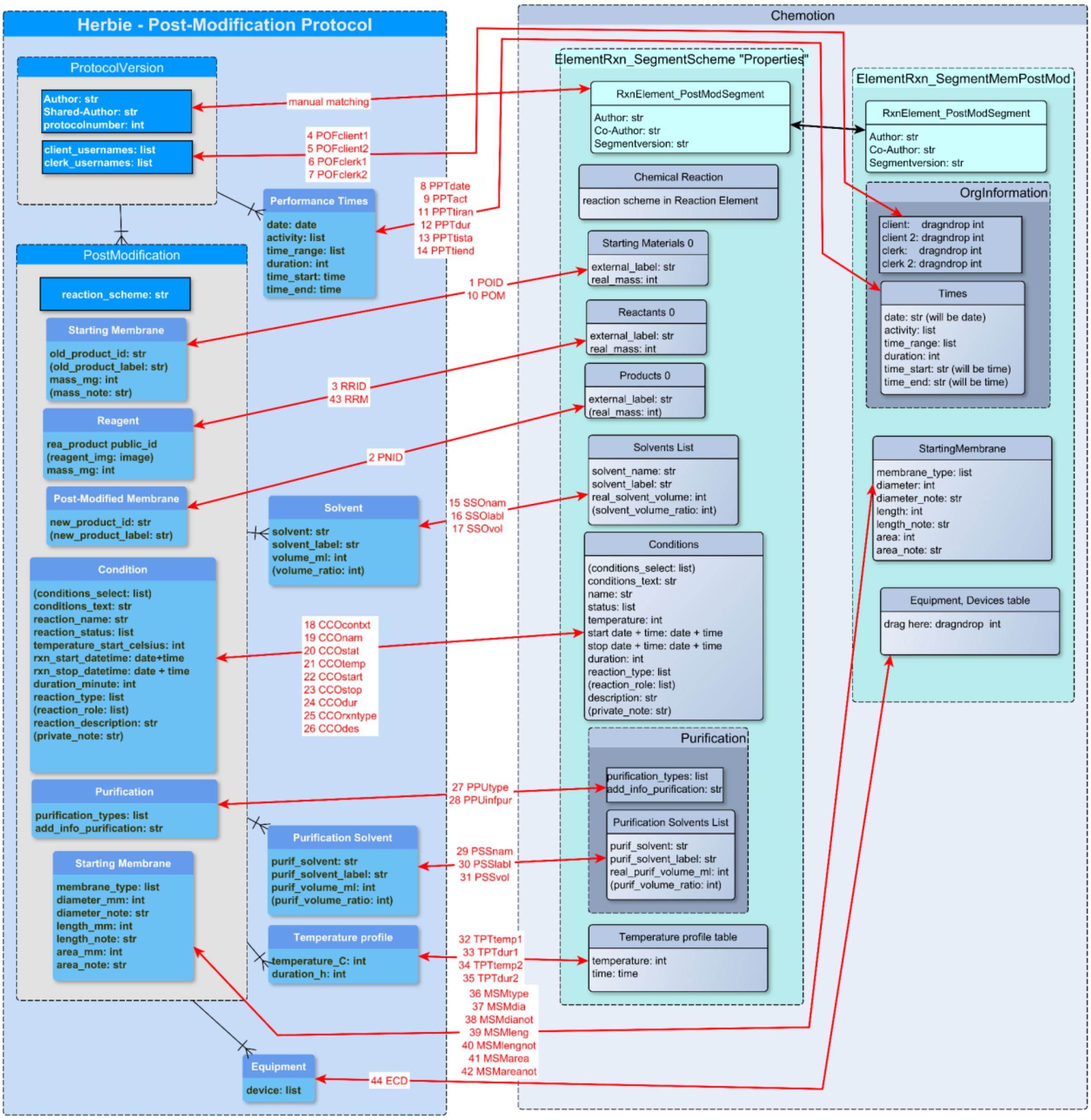

Figure S1. Mapping of ELN objects (input field variables) in the backend of Herbie and Chemotion for the use-case of chemical post-modification of PAN membranes. The mapping functions are typed in red.

Figure S1 presents a comprehensive mapping of all input fields (ELN objects) of Herbie's "post-modification" and Chemotion's "reaction" (including the generic segment "membrane post-modification"). Some objects cannot be mapped, set in parenthesis, as they are computed live, not accessible through the API or not present in both ELNs. Each of the ELNs'

corresponding objects are connected by a mapping function typed in red. If required, data types are converted by the Google Blockly transfer operator.

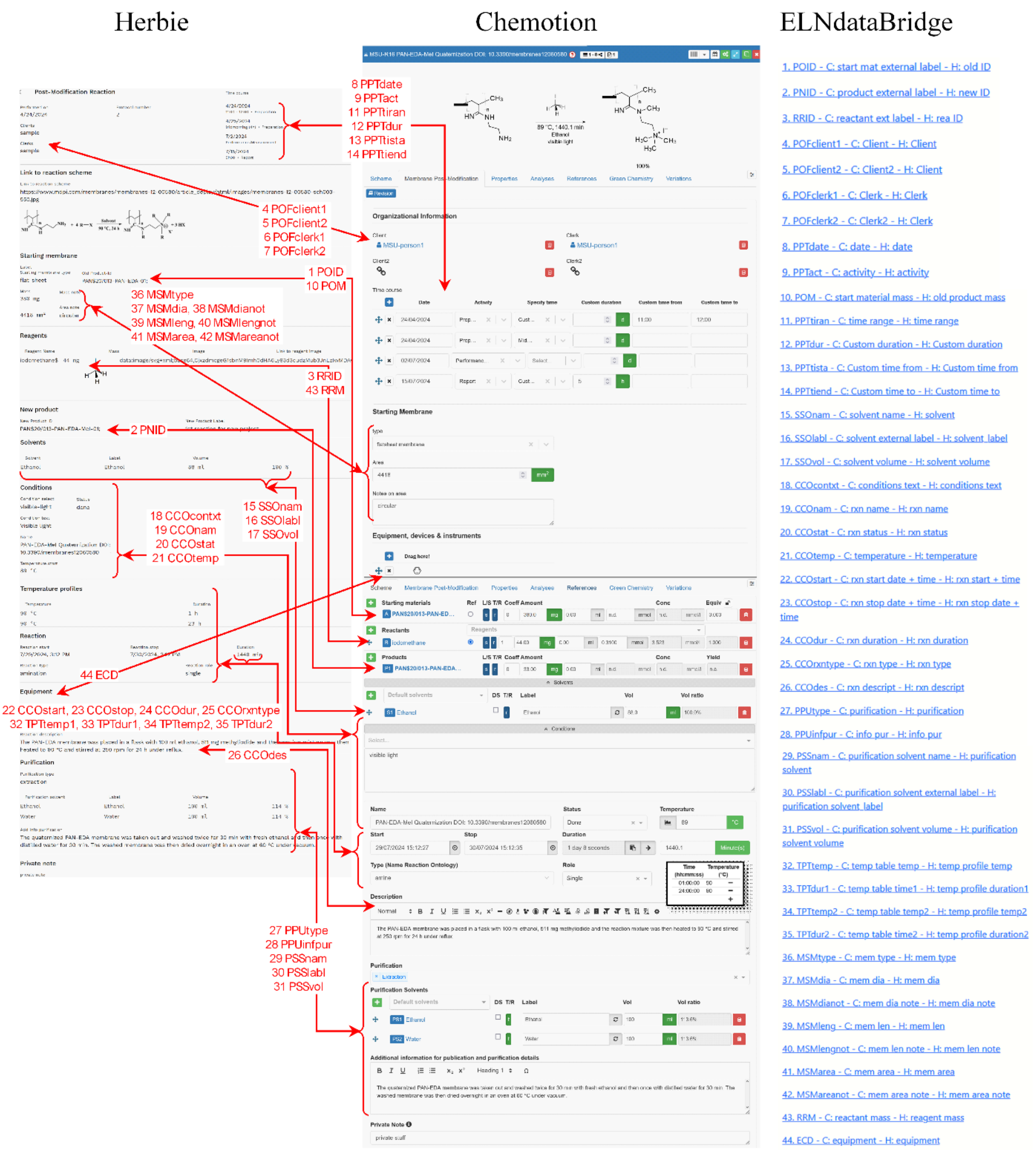

Figure S2. Mapping of the input fields in the frontend of Herbie and Chemotion for the use-case of chemical post-modification of PAN membranes. The mapping functions are typed in red, and in blue on the screenshot ELNdataBridge identifier list on the right side.

In coherence with the mapping of the backend variables (Figure S1), the mapping of frontend input fields highlights the high degree of completeness of mapping two ELN interfaces after

resolving all schematic conflicts. The mapping functions can then be used in the ELNdataBridge sync model as the identifier when listing the mapped objects.

2. User Interface

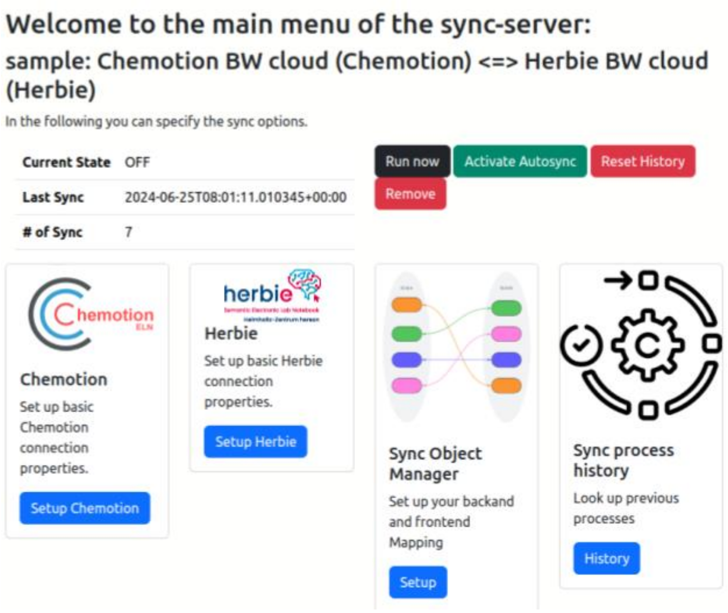

Figure S3. Main menu of the ELNdataBridge.

The menu is divided into three sections:

1. **Synchronisation Interface Information:** A brief overview of the interface at the top.
2. **Control Panel:** This provides insights into the frequency of interface execution, the last execution time, and whether it’s set to run automatically at regular intervals. The control panel includes, there are three action buttons:
    - Run Now: This button triggers the synchronisation interface immediately.
    - (De)Activate Autosync: This button enables or disables automatic synchronisation.
    - Reset History: This button can be used to delete the synchronisation history. This is useful, for example, to reset the assignment of element pairs and force a new pair search.
    - Remove: This button deletes the entire synchronisation interface.

3. **Setup Cards:** At the bottom of the menu, there’s a card-based settings menu. This menu contains four cards:
    - Connection Setup Cards: There are two cards dedicated to setting up the connections. These menus need to be adapted according to the requirements of the ELNs.
    - Sync Object Manager: This card menu is used to set up the actual mapping of the properties.
    - Sync Process History: This card menu allows users to view the logs of all previous synchronisation processes.

Chronologically, in the setup process, one starts from the bottom by setting up the connections, followed by setting up the mapping in the Sync Object Manager. The last step is running the sync in the central section. Sync process history, reset history, and synchronisation interface information have informative character.

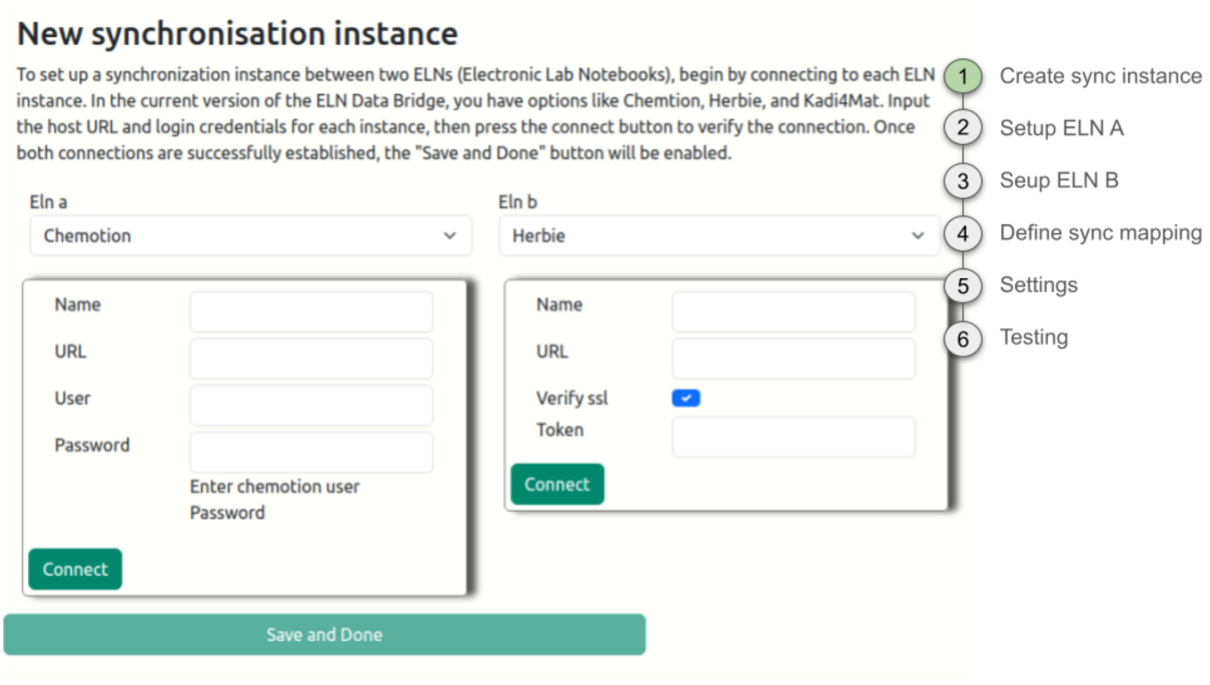

Figure S4. Full screenshot: Configuration of a synchronisation interface between local ELN instances of Chemotion and Herbie.

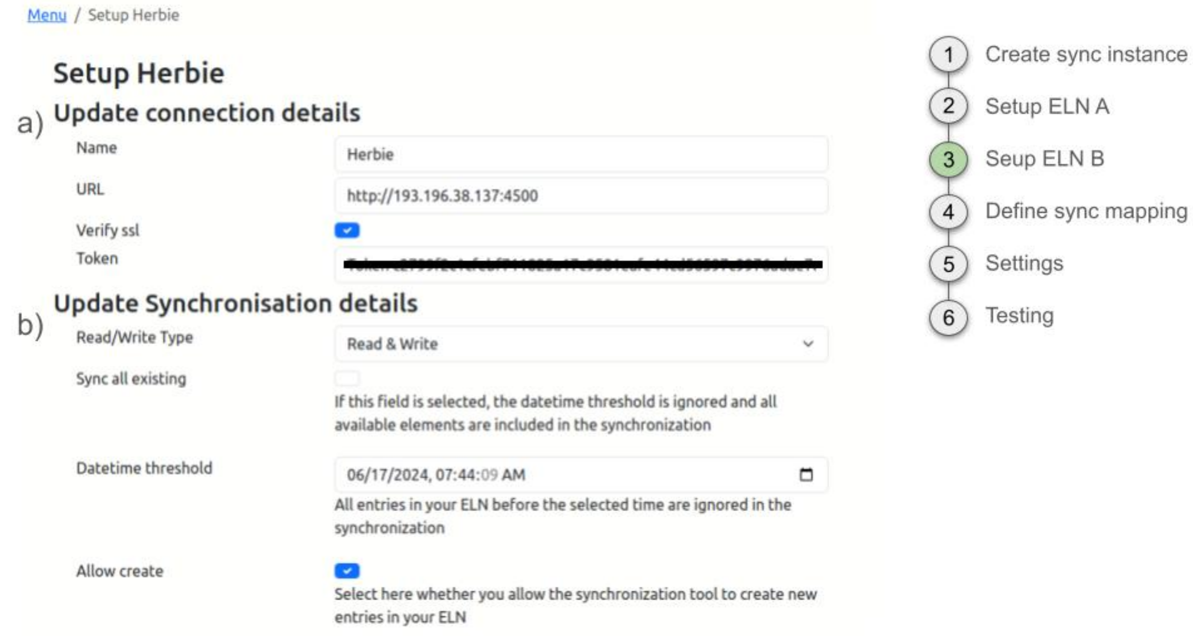

Figure S5. Settings of the synchronisation parameters for Herbie. a) Settings of the connection. b) Settings of the synchronisation behaviour: whether this platform should only be read or also written on, how existing elements should be handled and decided whether new elements need to be created.

3. Setup details

3.1. Details on building the pairing and mapping

An essential component for pairing of the ELNs and mapping of their input fields are the keys. The key fields are slightly more advanced due to the comprehensive and generic nature of the selection. The keys need to be selected from the JSON representation of the elements/objects displayed below the form. This representation provides a detailed insight into the internal structure of the elements/objects on both sides. By simply clicking on a key in the JSON, the entire key path up to that point will be automatically entered into the correct field. This feature ensures a smooth and efficient setup process. If these keys are not set, a new element will be created for each element to be synchronised that does not yet have a counterpart.

The order of the keys is only relevant if you have more than one synchronisation mapping which interact in some sense with each other. Then, the order of the key determines the sequence in which the single synchronisation mappings are processed. Thus, a synchronisation mapping with order 1 is processed before a synchronisation mapping with order 2.

3.2. Details on testing

Please note that no data is written when the test is executed!

In the dialog the JSON object is displayed but with the changes that result from the synchronisation process. Entries highlighted in yellow represent changes, while newly added fields are highlighted in green. Deleted entries are coloured red. Warnings are also displayed if the data type changes during synchronisation. If the value (as in Figure S6) “add_info_purification” was of type array in the original data set and is of type string after synchronisation, this means that it is very likely that writing the new value in the target ELN will generate an error. To fix this, the transfer operator needs to be adjusted.

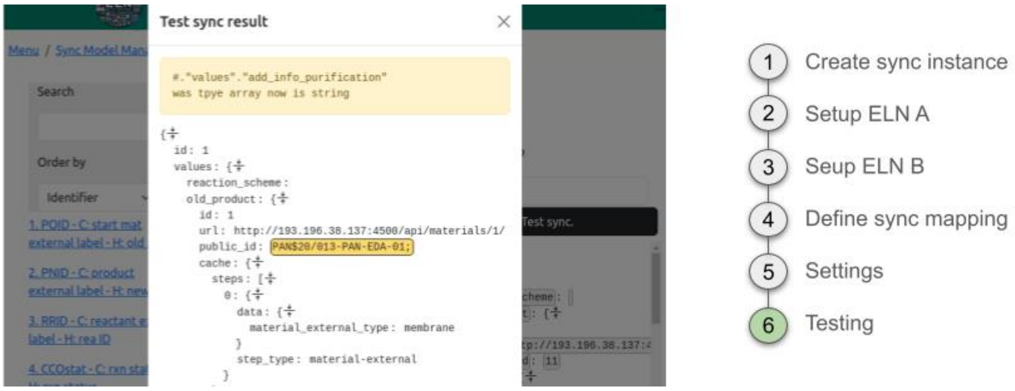

Figure S6. Test the mapping to see all the changes that would be caused by the mapping.

3.3. Details on synchronisation

When you click on the *Run now* button, the synchronisation process is started on the server and the browser is redirected to a log page (Figure S7) that shows the process history in real time with detailed logs of each change event (Figure S8).

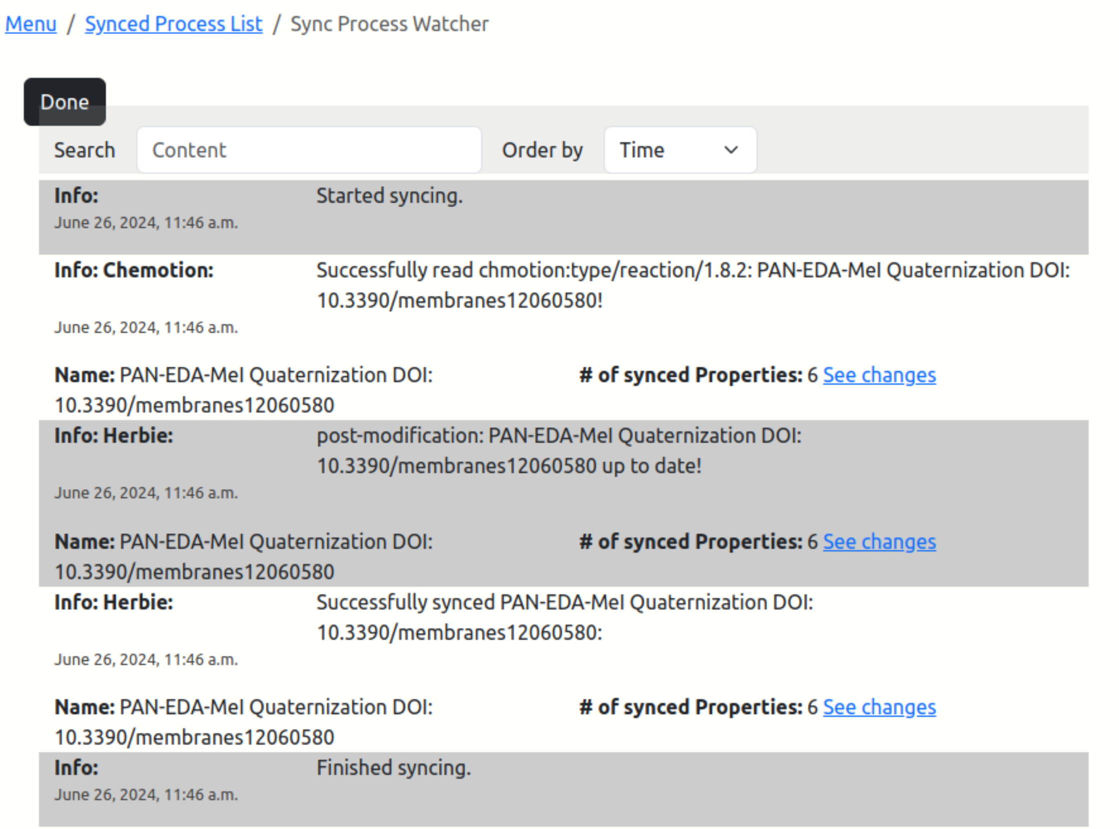

Figure S7. Synchronisation log to monitor the server activity and the history of all processes can be monitored in real time

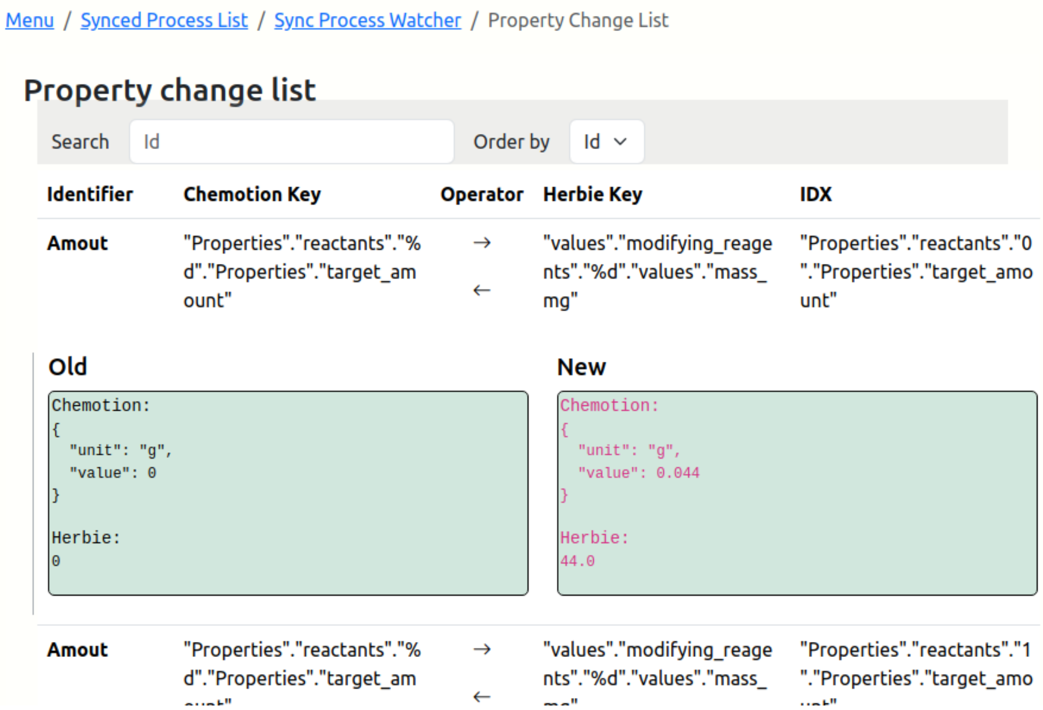

Figure S8. Detailed view of an incident, i.e. a change of an individual object, from the synchronisation log.

4. Dependencies arising from changes of the schemas

In the event of a schema change in one of the systems, the ability to synchronise is impaired. Such changes may include the removal, renaming or repositioning of fields within the schema. The extension of schemas may also need to be taken into account.

In order to resolve the issue, the ELNdataBridge simply requires the addition of a version tag to the models, for which the individual interface manager of the systems is responsible (see Figure S9).

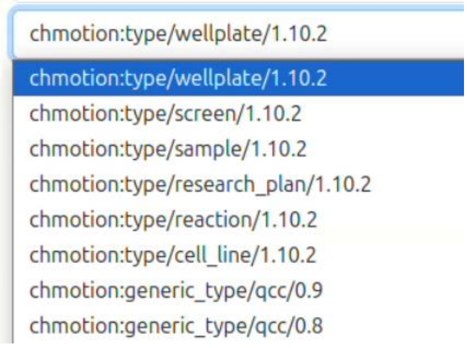

Figure S9. The version tag in Chemotion is added to the terminus of the model name. A distinction is made between generic and standard elements. The Chemotion version is utilised as the tag for standard models, while the incremental, user-defined version of the generic element is used for generic elements.

The version is incorporated into the model names in Chemotion, and consequently, a mapped object is only established for one particular version of a model. Following an update, a new mapping object must be created. This process is conducted by the system admin and is not automated at present. However, it is possible to simply copy the existing mapped object and select the new version of the model in the newly copied mapped object.

5. How to include a new ELN to ELNdataBridge

One key aspect of the ELNdataBridge system design is its effortless extension through the seamless integration of new ELNs, complete with a responsive API.

1. **Create a New Django App:** Begin by setting up a new Django application dedicated to the integration of the ELN.
2. **Design Connection Model and Form:** Develop a model and corresponding form to manage connections to the new ELN. This includes defining the necessary fields as for example username, password and/or token and validation rules.
3. **Implement Interface Manager:** Write an interface manager responsible for handling interactions between the ELNdataBridge and the new ELN Python API. This manager should facilitate communication and data exchange according to established protocols.
4. **Make Database Entry:** Incorporate functionality to register the new ELN within the ELNdataBridge system. This involves creating appropriate database entries to store relevant information about the ELN and its integration parameters.

A script included in the code allows points 1 and 4 to be completed automatically. A list of to dos is generated by executing the supplied script for the open points 2 and 3. More details are given on the Readme of the repository.

Bear in mind that the linking of entries must be done within the API translator. The latter is hard-coded, and no key under which this linked entry data could be found can be permanently set. For standard entries, such as samples or materials, this is not a problem since the identifiers are clear. For more generic entries, a solution has to be found. Moreover, the API translator will need to be supported in parallel with ELN version updates to ensure continuous integration with ELNdataBridge.

6. Classification of Open Source ELNs referring to the suitability for ELNdataBridge

In the Table S1, some of the currently available ELN solutions or software used as a kind of an ELN are listed and categorised according to their suitability to be used with ELNdataBridge. The information needed for this brief assessment was gained from ELN Finder[1] and direct feedback from the ELN providers. As the authors know the ELNs Chemotion and Herbie very well but are not experts for the functions and interfaces of other ELNs, therefore the table might contain inconsistencies and errors due to a non-sufficient understanding and interpretation of the ELNs' software. Although, the given table represents what we currently assess with the status available on 07/2024. Further changes within the ELNs' software might change the result of forthcoming assessments.

Table S1. Classification of existing open-source ELNs in terms of integrability into ELNdataBridge.

Name	Public API (yes/no)	Python API (Yes/No)	Type Characterisation (Yes/No)	Defined detailed structure[a]	Suitability[b] level for ELNdataBridge
Herbie	Yes	Yes	Yes	3	****
Chemotion	Yes	Yes	Yes	3	****
Kadi4Mat	Yes	Yes	Yes	2	***
ElabFTW	Yes	Yes	Yes	2	***
Sample DB	Yes	Yes	Yes	3	****
NOMAD ELN	No	Yes	Yes	3	****
OpenBis	Yes	Yes	Yes	2-3	***
Pasta	Yes	Yes	Yes	3	****
JuliaBase	Yes	Yes	Yes	3	****
Cheminfo	Yes	Yes	Yes	3	****
Sciformation	No	Yes	Yes	3	****
Open Enventory	No	No	Yes	3	x
LOGS-ELN	Yes	Yes	Yes	2	***
AI4Green	No	No	Yes	3	x
RSpace	Yes	Yes	Yes	2	****
MS OneNote	No	Yes	No	1	*

[a]1 = not structured, 2 = optional structured elements, 3 = fully structured; [b] Suitability was evaluated according to the information available on API accessibility, the characterisation of type, and the level of definition of the structure in detail. The higher the level gained, the more suitable the ELN for a successful application of ELNdataBridge.
**** = reading, writing, creating feasible with minimal effort
*** = reading with minimal effort, writing and creating feasible with higher effort
** = reading with higher effort, writing and creating impossible
* = reading with minimal effort, writing and creating impossible
x = not possible

1. Branschofsky, M. & Chudnov, D. DSpace. in *Proceedings of the second ACM/IEEE-CS joint conference on Digital libraries - JCDL '02* (ACM Press, New York, New York, USA, 2002). doi:10.1145/544317.544319.
